# Genome-wide identification and expression analysis of the *KCS* gene family in soybean (*Glycine max*) reveal their potential roles in response to abiotic stress

**DOI:** 10.3389/fpls.2023.1291731

**Published:** 2023-12-05

**Authors:** Yujie Gong, Deying Wang, Haojie Xie, Zewei Zhao, Yuyue Chen, Dongxue Zhang, Yexuan Jiao, Mengmeng Shi, Peng Lv, Qi Sha, Jing Yang, Pengfei Chu, Yongwang Sun

**Affiliations:** School of Agricultural Science and Engineering, Liaocheng University, Liaocheng, China

**Keywords:** very long chain fatty acids, 3-ketoacyl-CoA synthase, gene family, soybean, abiotic stress, expression profile

## Abstract

Very long chain fatty acids (VLCFAs) are fatty acids with chain lengths of 20 or more carbon atoms, which are the building blocks of various lipids that regulate developmental processes and plant stress responses. 3-ketoacyl-CoA synthase encoded by the *KCS* gene is the key rate-limiting enzyme in VLCFA biosynthesis, but the *KCS* gene family in soybean (*Glycine max*) has not been adequately studied thus far. In this study, 31 *KCS* genes (namely *GmKCS1* - *GmKCS31*) were identified in the soybean genome, which are unevenly distributed on 14 chromosomes. These *GmKCS* genes could be phylogenetically classified into seven groups. A total of 27 paralogous *GmKCS* gene pairs were identified with their Ka/Ks ratios indicating that they had undergone purifying selection during soybean genome expansion. *Cis*-acting element analysis revealed that *GmKCS* promoters contained multiple hormone- and stress-responsive elements, indicating that *GmKCS* gene expression levels may be regulated by various developmental and environmental stimuli. Expression profiles derived from RNA-seq data and qRT-PCR experiments indicated that *GmKCS* genes were diversely expressed in different organs/tissues, and many *GmKCS* genes were found to be differentially expressed in the leaves under cold, heat, salt, and drought stresses, suggesting their critical role in soybean resistance to abiotic stress. These results provide fundamental information about the soybean *KCS* genes and will aid in their further functional elucidation and exploitation.

## Introduction

Fatty acids with a hydrocarbon chain lengths of 20 or more carbon atoms are known as VLCFAs (Scott et al., 2022). The VLCFAs and their derivatives are the main components of numerous lipids that function in specific cell/tissue types, such as triacylglycerols that accumulate in the seeds, sphingolipids, and phospholipids that are essential for membrane homeostasis, cuticular waxes deposited on plant aerial surfaces, and suberin monomers that are indispensable for root development (Haslam and Kunst, 2013). Cuticular wax forms a hydrophobic layer covering most aerial plant surfaces. It plays a vital role in preventing excessive water loss, regulating transpiration, resistance against pathogen invasion, and tolerance against various environmental stresses (Kong et al., 2020; Arya et al., 2021; Batsale et al., 2021). The biosynthesis of VLCFAs is catalyzed by a fatty acid acyl-CoA elongase (FAE) multienzyme complex comprising four enzymes, including 3-ketoacyl-CoA synthase (KCS), β-ketoacyl-CoA reductase (KCR), β-hydroxy acyl-CoA dehydratase (HCD) and trans-2,3-enoyl-CoA reductase (ECR) (Bach and Faure, 2010; Kim et al., 2013). Using the plastid-generated C16 or C18 fatty acids as substrates, the endoplasmic reticulum-localized FAE sequentially adds two carbon units to a growing acyl chain through a repetitive cycle of condensation, keto-reduction, dehydration and enoyl-reduction (Kunst and Samuels, 2009). During the elongation process of VLCFAs, KCS catalyzes the condensation of C16 or C18 fatty acids with malonyl-CoA. Moreover, KCS exhibits strict substrate specificity and tissue specificity, and it is the key rate-limiting enzyme that determines the ultimate chain length and contents of VLCFAs (Shi et al., 2017; Ozseyhan et al., 2018). In contrast, the other three enzymes do not directly control VLCFA synthesis (Kunst and Samuels, 2009).

The first identified and characterized gene in the *KCS* family was *FAE1/AtKCS18*, specifically expressed in developing seeds, which catalyzes the synthesis of C20 and C22 fatty acids for energy storage in *Arabidopsis thaliana* (James et al., 1995; Rossak et al., 2001). Subsequently, numerous *KCS* genes have been cloned and functionally characterized in plants. The KCS proteins have two essential and conserved domains: the FAE1/Type III polyketide synthase-like protein domain (FAE1_CUT1_RppA) and the 3- Oxoacyl-[acyl-carrier-protein (ACP)] synthase III C-terminal domain (ACP_syn_III_C) (Sagar et al., 2015). *KCS* gene families have been identified in various higher plants, and the number of these genes varies greatly between species (Guo et al., 2016). For instance, 21 *KCS* genes were identified in *Arabidopsis thaliana* (Costaglioli et al., 2005; Joubès et al., 2008), 22 in rice (*Oryza sativa*; Yang et al., 2023), 25 in sorghum (*Sorghum bicolor*; Zhang et al., 2022a), 26 in maize (*Zea mays*; Campbell et al., 2019), 28 in apple (*Malus × domestica* Borkh.; Lian et al., 2020), 30 in peanut (*Arachis hypogaea*; Huai et al., 2020) and cabbage (*Brassica oleracea*; Xue et al., 2020), 33 in turnip (*Brassica rapa*; Xue et al., 2020) and barley (*Hordeum vulgare*; Tong et al., 2021), and as many as 58 in upland cotton (*Gossypium hirsutum*; Xiao et al., 2016) and rapeseed (*Brassica napus*; Xue et al., 2020).

The *KCS* gene family in *Arabidopsis thaliana* has been the most thoroughly studied and characterized in terms of physiological and molecular functions. It is divided into four classes (KCS1-like, FAE-like, FDH-like, CER6) or eight subclasses (α, β, γ, δ, ε, ζ, η, and θ) according to their phylogeny and duplication history (Costaglioli et al., 2005; Joubès et al., 2008). Previous studies have demonstrated that they catalyze the formation of diverse VLCFAs and play important roles in *Arabidopsis* developmental and metabolic processes, such as cuticular wax production (*AtKCS*s *1*, *2*, *3*, *4*, *5*, *6*, *9*, *16* and *20*) (Todd et al., 1999; Fiebig et al., 2000; Kim et al., 2013; Hegebarth et al., 2017; Kim et al., 2021; Huang et al., 2022; Huang et al., 2023), suberin metabolism in roots (*AtKCS*s *2*, *9* and *20*) (Franke et al., 2009; Lee et al., 2009; Kim et al., 2013), oil biosynthesis in seeds (*AtKCS18*) (James et al., 1995), and development of epidermis (*AtKCS10* and *AtKCS13*) (Gray et al., 2000; Pruitt et al., 2000). Moreover, several *AtKCS* genes have been confirmed to play crucial roles in abiotic stress responses and tolerance. For example, *AtKCS2* and *AtKCS20* expression levels were drastically elevated under various abiotic stresses, indicating that they may be involved in stress adaptation and tolerance (Lee et al., 2009; Lee and Suh, 2015a). *AtKCS1* was reported to be an important player involved in chilling tolerance (Chen et al., 2020c), and *AtKCS4* plays a key role in the regulation of lipid metabolism under heat and dark conditions (Luzarowska et al., 2023).

In other species, particularly in crop plants, numerous *KCS* genes have been demonstrated to be vital for developmental and stress tolerance mechanisms. *LeCER6* is an essential *KCS* gene that regulates fruit cuticular wax production in tomato (Smirnova et al., 2013). The maize *GL4*, a homolog of *AtKCS6*, is involved in cuticular wax accumulation in seedling leaves (Lee and Suh, 2015b). *HMS1* expression increased pollen viability under low moisture stress in rice (Chen et al., 2020b). Overexpression of *GhKCS13* enhanced the sensitivity to cold stress of transgenic cotton plants by modulating lipid and oxylipin biosynthesis (Wang et al., 2020a). Ectopic expression of *VvKCS11* from grape (*Vitis vinifera*) or *CcKCS6* from navel orange (*Citrus clementina*) enhanced the tolerance of transgenic *Arabidopsis* plants to various abiotic stresses (Guo et al., 2020; Yang et al., 2020). *MtKCS12*, predominantly expressed in the *Medicago truncatula* seed coat, controls the production of very long-chain lipid species in the seed coat and is essential for seed physical dormancy (Chai et al., 2021).

Soybean (*Glycine max*) is an economically important food and oil crop with high nutritional value, with its seed oil accounting for about 30% of all vegetable oil consumption worldwide (Zhan et al., 2020). It provides abundant nutrients for human nutrition, has various industrial uses, and is considered a suitable candidate crop for biodiesel production (Woyann et al., 2019). Moreover, soybean is one of the most commonly cultivated crops in arid and semi-arid areas where its growth and yield are usually hindered by various abiotic stresses, such as water deficiency, salt, and temperature extremes (Deshmukh et al., 2014; Gavili et al., 2018). To the best of our knowledge, there are limited studies regarding the soybean *KCS* gene family to date. As the *KCS* family genes are known to be crucial players in regulatory networks controlling biological and developmental processes as well as stress tolerance of plants, a thorough investigation of the *KCS* gene family in soybean is of great importance. In this study, 31 soybean *KCS* genes were identified using bioinformatics techniques. Their chromosomal distribution, gene structure, conserved motif distribution, gene synteny, and phylogenetic relationships were systematically analyzed. To further analyze the prospective functions of soybean’s *KCS* genes, their tissue-specific expression profiles were examined, as well as their response to cold, heat, salt, and drought stresses. These findings establish the groundwork for understanding the functions of the *GmKCS* gene family and offer crucial insights into their involvement in soybean’s stress tolerance mechanisms.

## Materials and methods

### Identification of the *KCS* genes in the genome of soybean

The Hidden Markov Models search tool (HMMER ver3.3.2; http://hmmer.org/) and Basic Local Alignment Search Tool for proteins (BLASTP; https://blast.ncbi.nlm.nih.gov/Blast.cgi) were used to retrieve and obtain the KCS sequences from the soybean genome (Wm82.a4.v1). The gff3, proteins, coding sequence, and genome files of soybean were downloaded from the phytozome database (https://phytozome-next.jgi.doe.gov/). The hidden Markov model (HMM) profiles of the two structural domains of KCS proteins, ACP_syn_III_C (Pfam: PF08541) and the FAE1_CUT1_RppA (Pfam: PF08392), were obtained from the Pfam database (http://pfam.xfam.org/). The 21 KCS protein sequences of Arabidopsis thaliana were downloaded from the Arabidopsis Information Resource (TAIR) database (https://www.arabidopsis.org/) and used for BLASTP comparisons against the soybean protein database. After integrating the results of HMMER and BLASTP searches, the non-redundant protein sequences were submitted to NCBI CD-search (https://www.ncbi.nlm.nih.gov/Structure/cdd/wrpsb.cgi) and the SMART server (https://smart.embl-heidelberg.de/) to investigate whether the two conserved domains are present. Proteins including both ACP_syn_III_C and the FAE1_CUT1_RppA domains were considered as *KCS* gene family members in soybean, and they were named according to their chromosomal location.

### Prediction of the physicochemical properties, subcellular localization, and TAH of GmKCS proteins

The ProtParam tool in the ExPASy website (https://web.expasy.org/protparam/) was used to predict the physical and chemical properties of GmKCS proteins, such as molecular weight, isoelectric point, instability index, aliphatic index, and the grand average of hydropathy index (GRAVY). The subcellular location and the transmembrane alpha-helix (TAH) of GmKCS proteins were predicted using the WoLF PSORT web server (https://wolfpsort.hgc.jp/) and the TMHMM website (https://services.healthtech.dtu.dk/service.php?TMHMM-2.0), respectively.

### Chromosomal localization and synteny analysis of *GmKCS* genes

The chromosome locations of GmKCS genes were retrieved from soybean genome annotation files. In the synteny analysis, all-against-all comparisons were performed in the soybean genome using BLASTP search with E-values less than 10^−10^ to obtain information on the gene pairs. Next, McScanX in TBtools (Chen et al., 2020a) was employed based on the BLASTP results to analyze synthetic blocks and gene duplication events. A circular map of GmKCS gene pairs was drawn using the Advanced Circos plugin in TBtools. Duplicated *GmKCS* gene pairs were displayed by connected red solid lines. The Ka and Ks values of the duplicated *GmKCS* gene pairs were calculated using the Ka/Ks Calculator in TBtools to analyze the divergence of duplicated genes. The duplication time was calculated according to a published method using the following formula: Time = Ks/(2 × 6.1 × 10^−9^) (Fan et al., 2013).

### Phylogenetic analysis of KCS proteins

Alignment and phylogenetic analysis of KCS protein sequences from Arabidopsis (Joubès et al., 2008), turnip (Xue et al., 2020), soybean, rice (Yang et al., 2023) and sorghum (Zhang et al., 2022a) was performed using the MEGA 11.0 software (https://www.megasoftware.net/). The aligned sequences were subjected to neighbor-joining (NJ) tree construction with 1000 bootstrap replications, pairwise deletions, based on the Poisson model (Kumar et al., 2016).

### Analysis of gene structure, *cis*-acting elements and conserved motif of *GmKCS* genes

The gff3 file of soybean was used to analyze the nucleotide sequence structure of *GmKCS* genes. The exon-intron distribution chart was generated using the TBtools software. The 2.0 kb upstream sequence from each *GmKCS* gene was selected as the promoter region and extracted from the soybean genome. The PlantCARE website (https://bioinformatics.psb.ugent.be/webtools/plantcare/html/) was used to predict the cis-acting elements. The functions and sequences of putative cis-acting elements of *GmKCS* genes were categorized into hormone- and stress-responsive elements. Conserved motifs of the GmKCS proteins were identified using the Multiple Expectation Maximization for Motif Elicitation (MEME) online website (http://meme-suite.org/) with the following parameters: a maximum number of 10 motifs and an optimum motif width of 6 - 200 amino acid residues. TBtools software was used to visualize the conserved motifs.

### Expression pattern of *GmKCS* genes in various tissues/organs

RNA-seq data from nine tissues/organs of soybean were downloaded from the Phytozome database, including the roots, root hairs, nodules, stems, leaves, shoot apical meristem (SAM), pods, and seeds. The expression levels of each gene were represented by its fragments per kilobase of exon per million fragments mapped (FPKM) values, and a heatmap showing tissues-specific expression profiles was generated using the log_2_-transformed (FPKM + 1) values of *GmKCS* genes in the TBtools software.

### Plant growth and stress treatments of soybean seedlings

The seeds of soybean cultivar Williams 82 were germinated on absorbent paper for 3 days and then sown on a planting tray for culture with 1/2 Hoagland nutrient solution under a 16-h-light/8-h-dark photoperiod at 25°C/18°C (day/night). After growing to the opening of the first leaf (V1 stage, Fehr and Caviness, 1977), plants with consistent growth were selected and subjected to four abiotic stress treatments. For cold and heat stress, the seedlings were exposed to 4°C and 37°C, respectively (Liu et al., 2015; Wang et al., 2016b). For salt stress, the seedlings were cultured in a 150 mM NaCl solution (Hu et al., 2022). For drought stress, the seedlings were hydroponically cultured in a 20% PEG6000 (w/v) solution (Wang et al., 2021). The control group was cultured in a 1/2 Hoagland nutrient solution. After 24 hours of treatment, the soybean leaves were sampled separately and frozen immediately in liquid nitrogen, then stored at –80°C until RNA extraction. Each sample was collected from five individual plants, and the biological assays were repeated independently three times.

### RNA extraction, cDNA synthesis, and qRT-PCR analysis

Total RNA was extracted from leaves at different plant growth stages under the stress and control treatments using an RNA plant extraction kit (TSINGKE, cat.TSP401, China) according to the manufacturer’s instructions. Briefly, leaves were ground into fine powder in liquid nitrogen, and then 100 mg of powder was transferred to 0.45 µl Buffer RL. The RNA was purified using the RNase-Free Columns CS and finally eluted in 30 µl of RNase-free ddH_2_O. One microgram of RNA was reverse-transcribed to generate the first-strand cDNA using a Prime Script RT reagent kit (TSINGKE, cat. TSK301S, China) and stored at –20 °C until use. The quantitative real-time polymerase chain reaction (qRT-PCR) was carried out for each sample using a SYBR Green qRT-PCR kit (TSINGKE, cat.TSE201, China) with a LightCycler480 instrument (Roche). The qRT-PCR amplification program used was set as follows: stage 1, initial denaturation at 95°C for 30 s; stage 2, 40 cycles of 95°C for 5 s (denaturation), 60°C for 30 s (annealing); stage 3, melting curve. Relative quantification was performed using the 2^−ΔΔCt^ method (Derveaux et al., 2010) using the *GmTub* gene (*Glyma.05G157300*, Wang et al., 2016a) as the internal control. The data were analyzed and illustrated using Graphpad Prism 8.0 (https://www.graphpad.com/). The values were the average from three technical measurements, and the data were presented as mean ± standard error (SE). Gene-specific primers used in this study are listed in **Supplementary Table S1**. Statistical significance of the data was analyzed using independent-samples t-test. Error bars indicate SE and p-value < 0.05 (*) or < 0.01 (**).

## Results

### Identification of *KCS* genes in soybean and characterization of their protein physicochemical properties

**Figure 1:**
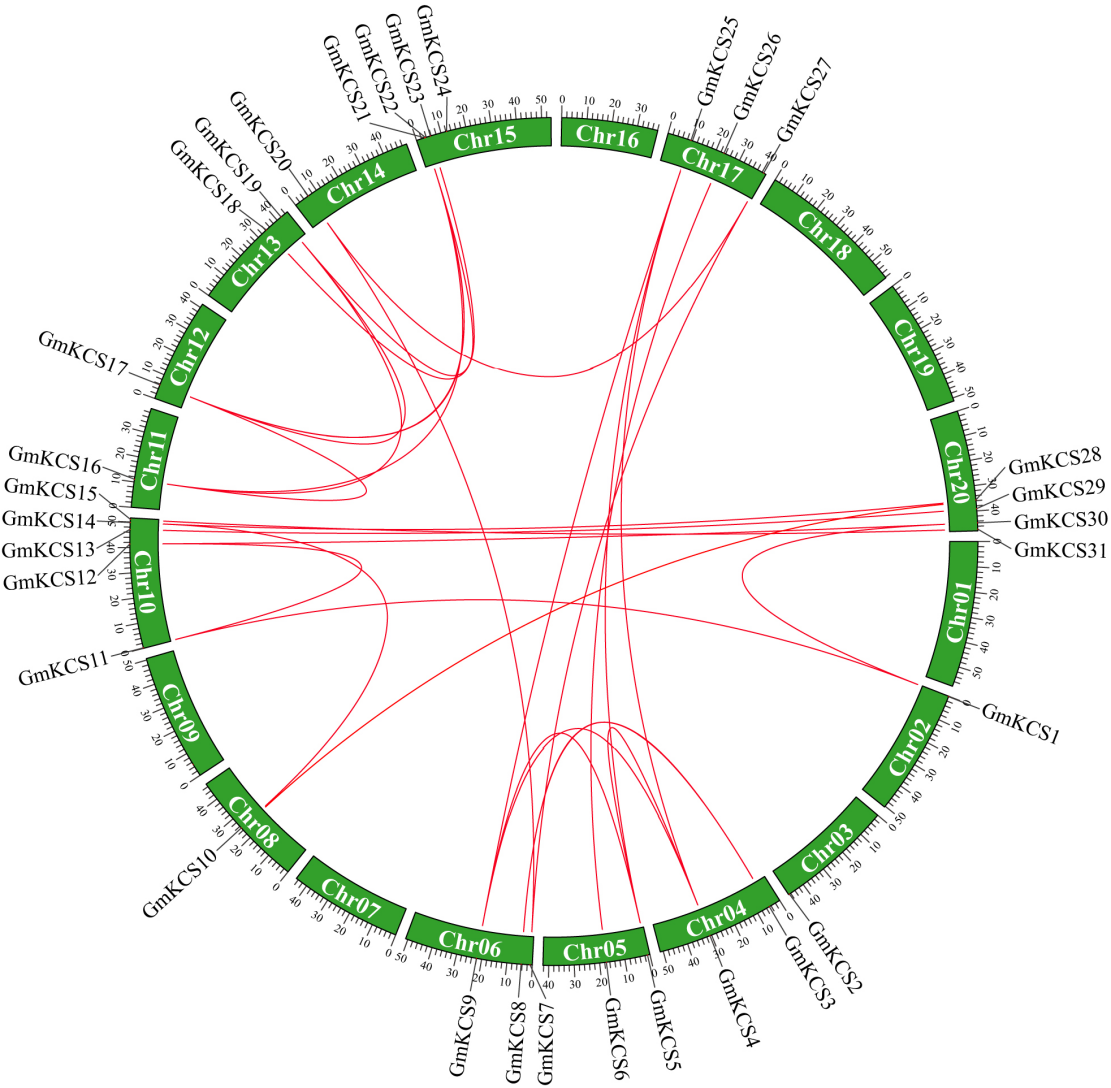
Chromosome location and the syntenic relationships among *GmKCS* genes. The positions of the *GmKCS* genes on the chromosomes are shown on the outside. The green colored boxes indicate the different chromosomes (Chr1-Chr20) of soybean. The red lines connecting genes from different chromosomes represent 27 segmental duplication events among the *GmKCS* genes.

After integrating the search results of HMMER and BLASTP, 56 non-redundant proteins were obtained and subjected to conservative domain identification (**Supplementary Table S2**). A total of 31 proteins were found to contain both the ACP_syn_III_C and the FAE1_CUT1_RppA domains, and they were considered as *KCS* gene family members of soybean. The corresponding genes were designated as *GmKCS1* to *GmKCS31* according to their positions on soybean chromosomes (**Table 1**; **Figure 1**). The nucleotide sequence length of *GmKCS* genes varied from 1332 bp (*GmKCS7*) to 6891 bp (*GmKCS6*), and their coding sequence ranged from 1332 bp (*GmKCS7*) to 1611 bp (*GmKCS3*). Furthermore, the physicochemical properties of the GmKCS proteins were characterized. GmKCS7 and GmKCS3 were the shortest and the longest GmKCS proteins, containing 443 and 536 amino acid residues, respectively. The GmKCS protein molecular weight ranged from 49.65 kDa (GmKCS11) to 60.95 (GmKCS3) kDa, and the theoretical isoelectric point ranged from 8.49 (GmKCS17) to 9.40 (GmKCS12), indicating that all GmKCS proteins tend to be weakly basic. Analysis of the instability index indicated that nine proteins are potentially unstable (> 40), while the remaining 22 are probably stable proteins (ranging from 31.70 to 39.88). The aliphatic index, which ranged from 86.54 (GmKCS8) to 103.78 (GmKCS29), indicated the thermal stability of GmKCS proteins. The GRAVY values of GmKCS proteins ranged from -0.228 (GmKCS3) to 0.093 (GmKCS29), suggesting that they are amphoteric proteins (**Table 1**).

**Table 1:** Characterization of the *GmKCS* genes and GmKCS proteins.

Name	ID	Gene Length (bp)	Protein Length (aa)	Molecular Weight (kDa)	Isoelectric Point	Instability Index	Aliphatic Index	GRAVY
*GmKCS1*	*Glyma.02G001500*	2844	521	58.31	9.25	34.23	93.97	-0.042
*GmKCS2*	*Glyma.03G260300*	1593	530	58.95	9.10	39.60	90.00	-0.136
*GmKCS3*	*Glyma.04G057800*	4907	536	60.95	9.18	41.12	86.57	-0.228
*GmKCS4*	*Glyma.04G149300*	1533	510	57.35	9.21	35.74	91.76	-0.103
*GmKCS5*	*Glyma.05G011100*	1533	510	57.32	9.22	38.18	93.88	-0.102
*GmKCS6*	*Glyma.05G083000*	6891	469	53.09	8.91	33.87	93.50	-0.045
*GmKCS7*	*Glyma.06G012500*	1332	443	49.65	9.33	31.70	93.75	0.026
*GmKCS8*	*Glyma.06G058500*	4625	535	60.71	9.23	41.19	86.54	-0.214
*GmKCS9*	*Glyma.06G214800*	1533	510	57.31	9.17	35.67	93.49	-0.066
*GmKCS10*	*Glyma.08G261100*	1491	496	56.11	9.38	39.28	99.48	-0.017
*GmKCS11*	*Glyma.10G001800*	1879	517	58.08	9.40	36.04	92.61	-0.041
*GmKCS12*	*Glyma.10G179400*	2279	517	57.99	9.11	40.72	93.54	-0.033
*GmKCS13*	*Glyma.10G241700*	1446	481	53.79	8.57	39.74	99.90	0.037
*GmKCS14*	*Glyma.10G274400*	1491	496	56.09	9.09	38.78	100.06	0.024
*GmKCS15*	*Glyma.10G291700*	1452	483	55.01	8.96	35.97	88.41	-0.107
*GmKCS16*	*Glyma.11G144809*	1539	512	58.05	8.68	44.64	91.45	-0.085
*GmKCS17*	*Glyma.12G075100*	1416	471	53.29	8.49	43.34	89.04	-0.157
*GmKCS18*	*Glyma.13G238600*	2252	509	57.55	9.11	32.98	93.50	-0.122
*GmKCS19*	*Glyma.13G331600*	1422	473	53.48	8.85	38.82	90.30	-0.191
*GmKCS20*	*Glyma.14G074300*	1533	510	57.11	9.03	41.67	100.16	0.035
*GmKCS21*	*Glyma.15G042500*	1413	470	53.16	8.91	38.69	88.57	-0.198
*GmKCS22*	*Glyma.15G046300*	1380	459	51.44	8.73	39.03	96.19	-0.007
*GmKCS23*	*Glyma.15G074700*	2204	509	57.56	9.07	33.59	94.83	-0.106
*GmKCS24*	*Glyma.15G149400*	1377	458	51.47	8.96	44.56	94.54	0.040
*GmKCS25*	*Glyma.17G118700*	1533	510	57.49	9.31	38.72	94.65	-0.097
*GmKCS26*	*Glyma.17G183700*	6450	467	52.75	8.67	33.60	95.57	-0.031
*GmKCS27*	*Glyma.17G251000*	1542	513	57.41	9.19	39.88	100.16	0.029
*GmKCS28*	*Glyma.20G115500*	1491	496	56.06	9.18	39.29	101.03	0.026
*GmKCS29*	*Glyma.20G152500*	1536	511	57.81	8.57	41.13	103.78	0.093
*GmKCS30*	*Glyma.20G210900*	2261	517	57.93	9.10	40.44	93.54	-0.028
*GmKCS31*	*Glyma.20G240900*	1458	485	55.16	8.80	37.23	90.45	-0.069

### Subcellular localization and TAH domains of GmKCS proteins

The subcellular localization prediction analysis revealed that GmKCS proteins were mainly localized in the plasma membrane, endoplasmic reticulum, chloroplast, and vacuole (**Supplementary Figure S1**), which suggested that GmKCS proteins might be mostly expressed and function in these organelles. The KCS proteins have been reported to have transmembrane localization, and their transmembrane-spanning domains are necessary for their biological function (Ghanevati and Jaworski, 2002). Prediction of the TAH domains of GmKCS proteins suggested that most of them (14, 45.16%) had 2 TAHs, 7 had 3 TAHs, 5 had 1 TAH, and GmKCS13/17/21/22/29 had no TAH domains (**Supplementary Figure S2**). Most TAH domains of the GmKCS proteins are located in the 100 amino acid residues of the N-terminus, and only a few are located in the middle of the protein sequence (**Supplementary Figure S2**).

### Chromosomal distribution and duplication analysis of *GmKCS* genes

To map the chromosomal distribution of *GmKCS* genes, their physical location on the chromosomes was investigated. As shown in **Figure 1**, *GmKCS* genes were unevenly distributed on 14 of the 20 soybean chromosomes. Chr10 had the highest number of *GmKCS* genes (5), followed by Chr15 (4) and Chr20 (4), Chr6 (3) and Chr17 (3). Each of Chr4, Chr5, and Chr13 had 2 *GmKCS* genes, and only one *GmKCS* gene was located on each of Chr2, Chr3, Chr8, Chr11, Chr12, and Chr14. It is well-accepted that segmental and tandem duplications are two important mechanisms driving the expansion of gene families (Lynch and Conery, 2000). To further investigate the gene duplication events within the *GmKCS* family, a collinearity relationship analysis was performed. Twenty-seven pairs of segmentally duplicated genes were identified (**Figure 1**). The nonsynonymous (*K*a) and synonymous (*K*s) substitution ratios (*K*a/*K*s) were calculated to evaluate the selection pressure on *GmKCS* gene duplications. The *K*a/*K*s ratios for the 27 duplicated pairs ranged from 0.03 to 0.29 (**Supplementary Table S3**), implying that *GmKCS* genes evolved under strong purifying selection. Furthermore, it was estimated that the duplication events in the *GmKCS* gene family might have occurred 8.80 to 143.53 million years ago (Mya) (**Supplementary Table S3**).

### Phylogenetic analysis of *GmKCS* genes

**Figure 2:**
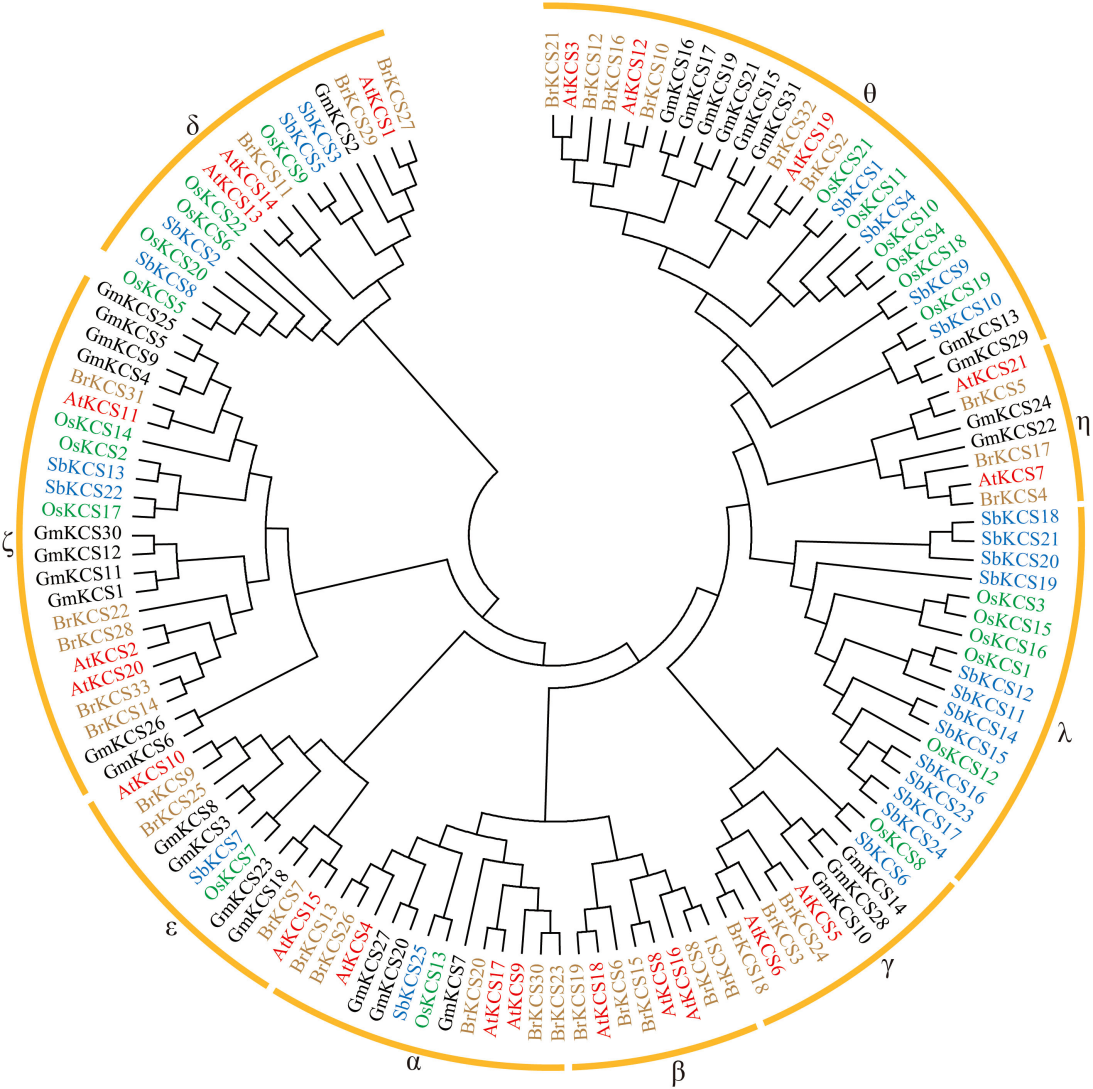
Phylogenetic analysis of KCS proteins from *Glycine max* (*Gm*) and *Arabidopsis thaliana* (*At*). 21 AtKCS and 31 GmKCS were aligned with the Clustal W program, and a neighbor-joining phylogenetic tree with 1000 bootstrap replications was constructed using the MEGA 11.0 software.

To elucidate the evolutionary relationships of the *KCS* genes, a total of 131 KCS protein sequences from five species were used to construct a phylogenetic tree, inlcuding 21 from *Arabidopsis*, 33 from turnip, 31 from soybean, 21 from rice, and 25 from sorghum (**Supplementary Data Sheet 1**). As shown in **Figure 2** and **Table 2**, these KCSs were divided into nine groups (α-θ), which were named based on the eight *Arabidopsis* KCS subclasses (Joubès et al., 2008). Interestingly, group β only existed in in two cruciferous plants, *Arabidopsis* and turnip. Groups α, γ, δ, ε, ζ and θ existed in all five species, while group λ and η only existed in monocotyledonous and dicotyledonous plants, respectively. GmKCS proteins were disproportionally distributed in seven groups except for groups β and λ. Group ζ contained the highest number (10) of GmKCSs, followed by group θ (8), ϵ (4), α and γ (3 each), η (2), and δ (1) (**Figure 2**; **Table 2**).

**Table 2:** The total number of *KCS* genes in each group of *Arabidopsis*, turnip, soybean, rice and sorghum.

Group	Arabidopsis	Turnip	Soybean	Rice	Sorghum
α	3	5	3	1	1
β	3	5	0	0	0
γ	2	3	3	1	1
δ	3	3	1	4	4
ε	2	3	4	1	1
λ	0	0	0	5	12
ζ	3	5	10	3	2
η	2	3	2	0	0
θ	3	6	8	6	4
Total	21	33	31	21	25

### Gene structure and motif composition of GmKCSs

**Figure 3:**
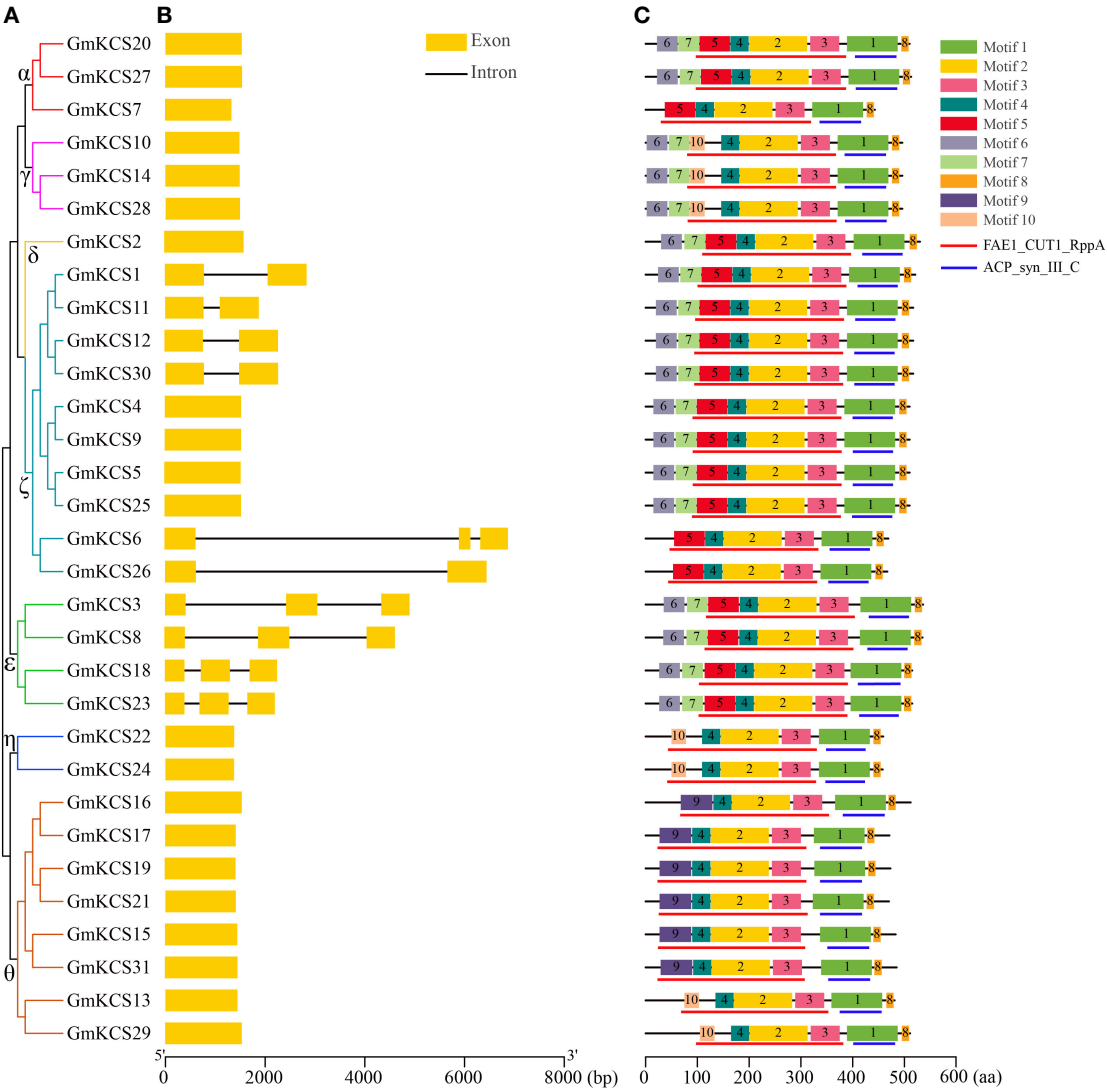
Schematic diagrams of *GmKCS* genes and motif composition of GmKCS proteins. **(A)** A rootless neighbor-joining tree was constructed based on the complete sequences of the 31 GmKCS proteins using the MEGA11.0 software. **(B)** Structural analyses of the exon-intron boundaries of the *GmKCS* genes with the yellow boxes and the black lines indicating the exons and introns, respectively. **(C)** Distribution map of the conserved motifs in GmKCS proteins. The ten putative motifs and the FAE1_CUT1_RppA and ACP_syn_III_C domains are represented by different colored boxes and red and blue lines, respectively. The length of the exon-intron junctions and amino acid sequences is inferred by the ruler at the bottom.

To analyze the structural composition of *GmKCS* genes, their exon-intron arrangements were obtained by comparing each gene’s coding sequence and DNA sequences. The results showed that most *GmKCS* genes were relatively simple in structure, e.g., the number of introns ranged from 0 to 3. Most *GmKCS* genes (21, 67.74%) contained no introns in their open reading frame sequences. Five genes (*GmKCS*s *1*, *11*, *12*, *26*, and *30*) from group ζ contained one intron, four genes (*GmKCS*s *3*, *8*, *19*, and *24*) from group ϵ and one (*GmKCS6*) from group ζ contained two introns (**Figures 3A, B**). Motif analysis was conducted to identify the conserved features of GmKCS proteins, with ten conserved motifs identified, ranging from 15 to 113 amino acid residues in length (**Figure 3C**; **Supplementary Figure S3**). Motifs 1, 2, 3, 4, and 8 were present in all GmKCS proteins, among which motif 1 corresponds to the ACP_syn_III_C domain, while motifs 2, 3, and 4 correspond to a part of the FAE1_CUT1_RppA domain. Although motifs 5, 9, and 10 were present in different GmKCS proteins, they were also included in the FAE1_CUT1_RppA domain, indicating that sequence variation has occurred in the conserved domains of GmKCS proteins. Motifs 6 and 7 were present in the N-terminus of most group α, γ, δ, ζ, and ε GmKCS proteins (except GmKCSs 6, 7, and 26) but were absent in the members of the other two groups (**Figure 3C**; **Supplementary Figure S3**).

### Distribution of cis-acting elements in *GmKCS* gene promoters

**Figure 4:**
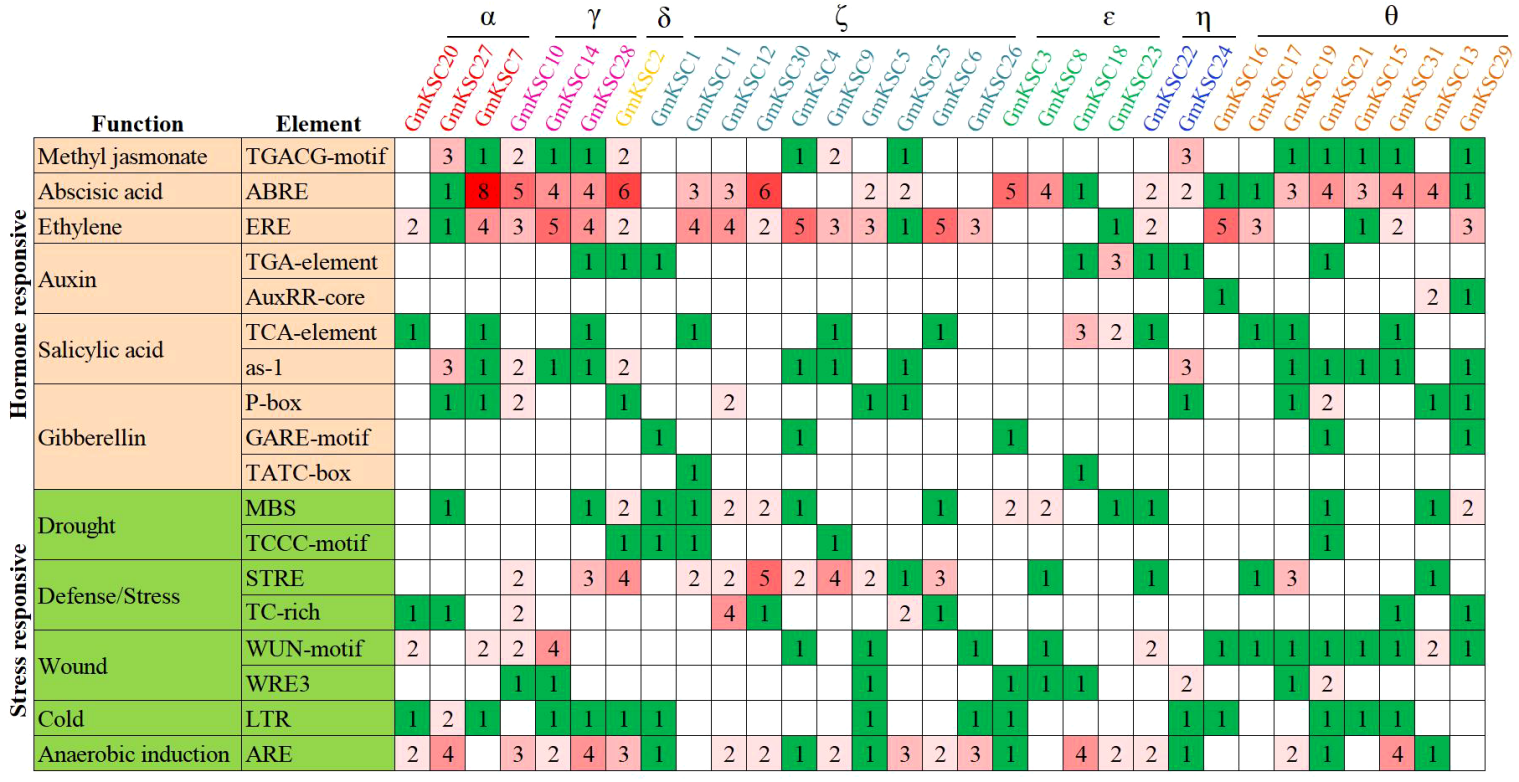
*Cis*-acting elements in the promoter regions of the 31 *GmKCS* genes. The color and number of the grid indicated numbers of different *cis*-acting elements in *GmKCS* genes.

To explore the potential regulatory cues of gene’s expression, 1.5 - 2.0 kb sequences upstream of the start codon were often considered as its promoter sequence and used for *cis*-acting elements analysis (Lian et al., 2020; Zhang et al., 2022a; Sun et al., 2023; Yang et al., 2023). In this study, 2.0 kb sequences upstream of the start codon of *GmKCS* genes were extracted and submitted to the PlantCARE website. A total of 18 *cis*-acting elements that related to hormone- and stress-responsiveness were obtained (**Figure 4**). Among the hormone-responsive elements, the number of abscisic acid-responsive elements was the highest (79), followed by the ethylene- (68) and salicylic acid-responsive elements (36). *Cis*-acting elements involved in abscisic acid-, ethylene-, and salicylic acid-responsiveness were found in the promoter of 25, 23, and 22 *GmKCS* genes, respectively. The promoters of 11, 15, and 17 *GmKCS* genes contain auxin-, methyl jasmonate-, and gibberellin-responsive elements, respectively. All *GmKCS* promoters contained at least one type of hormone-responsive element, and *GmKCS29* contained all the hormone-responsive elements mentioned above. Five stress-responsive elements were identified in *GmKCS* promoters. Among them, the number of anaerobic induction-responsive elements was the highest (53), followed by defense/stress- (51), wound- (36), drought- (27), and cold-responsive elements (16). These elements were found in 24, 20, 20, 17, and 15 *GmKCS* promoters, respectively. All *GmKCS* promoters contained at least two types of stress-responsive elements (**Figure 4**).

### Expression profiles of *GmKCS* genes in different tissues of soybean

**Figure 5:**
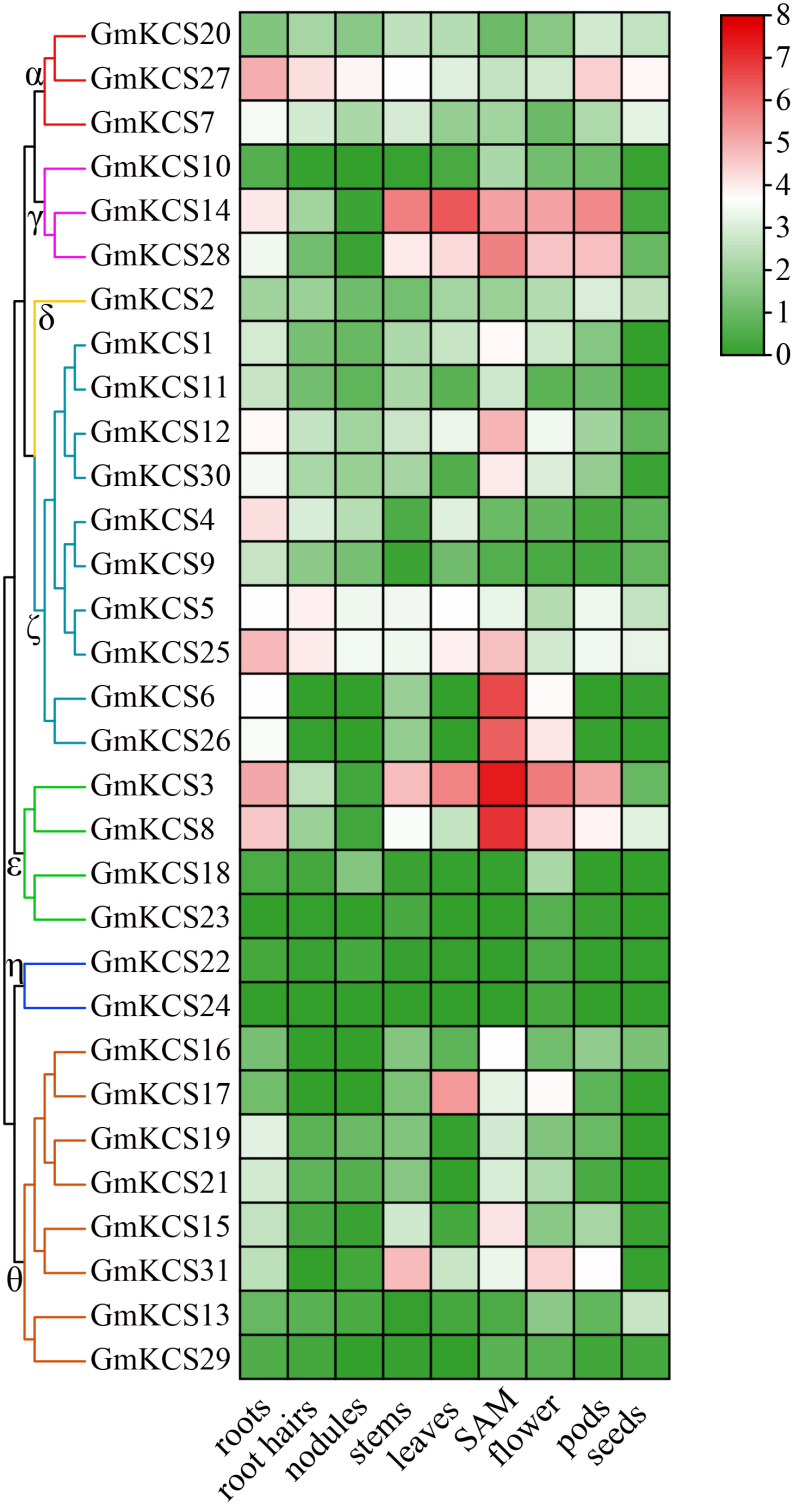
Expression profiles of the *GmKCS* genes. The transcription levels of *GmKCS* genes in nine selected tissues or organs of soybean plants were analyzed based on data collected from Phytozome. A heatmap was generated using TBtools. The color scale from blue to red indicates the increased expression levels of the genes.

To further investigate and elucidate the roles of *GmKCS* genes in soybean development, their expression profiles were analyzed in nine soybean tissues/organs based on published data collected from the Phytozome database. The gene expression values are represented by FPKM. Genes with FPKM values lower than 1.00 were considered to be not expressed, and the others were considered as low (1.00 ≤ FPKM < 5.00), moderate (5.00 ≤ FPKM < 15.00), and highly (FPKM ≥ 15.00) expressed genes (Girard et al., 2017). As shown in **Figure 5** and **Supplementary Table S4**, *GmKCS* genes exhibited different expression profiles among the nine soybean tissues/organs, regardless of whether they belonged to the same group. Except for *GmKCS*s *22*, *23*, *24*, and *29*, the other 27 *GmKCS* genes are expressed in at least one of the nine tissues/organs. *GmKCS*s *2*, *5*, *7*, *20*, *25*, and *27* were expressed at different levels throughout the soybean plant, indicating that they could be widely involved in multiple developmental processes in soybean. *GmKCS*s *3*, *4*, *8*, *14*, *25*, and *27* were highly expressed in the roots, and *GmKCS*s *3*, *14*, and *28* were expressed at high levels in all aerial plant parts except for the seeds, suggesting that these genes play a critical role in soybean vegetative growth. Several genes are expressed predominantly in specific tissues/organs, such as *GmKCS*s *6*, *15*, and *26*, expressed explicitly in the SAM, and *GmKCS17* expressed in the leaves, implying that these genes play an indispensable role in tissue-specific developmental and metabolic processes.

### Expression of *GmKCS* genes in response to abiotic stress

**Figure 6:**
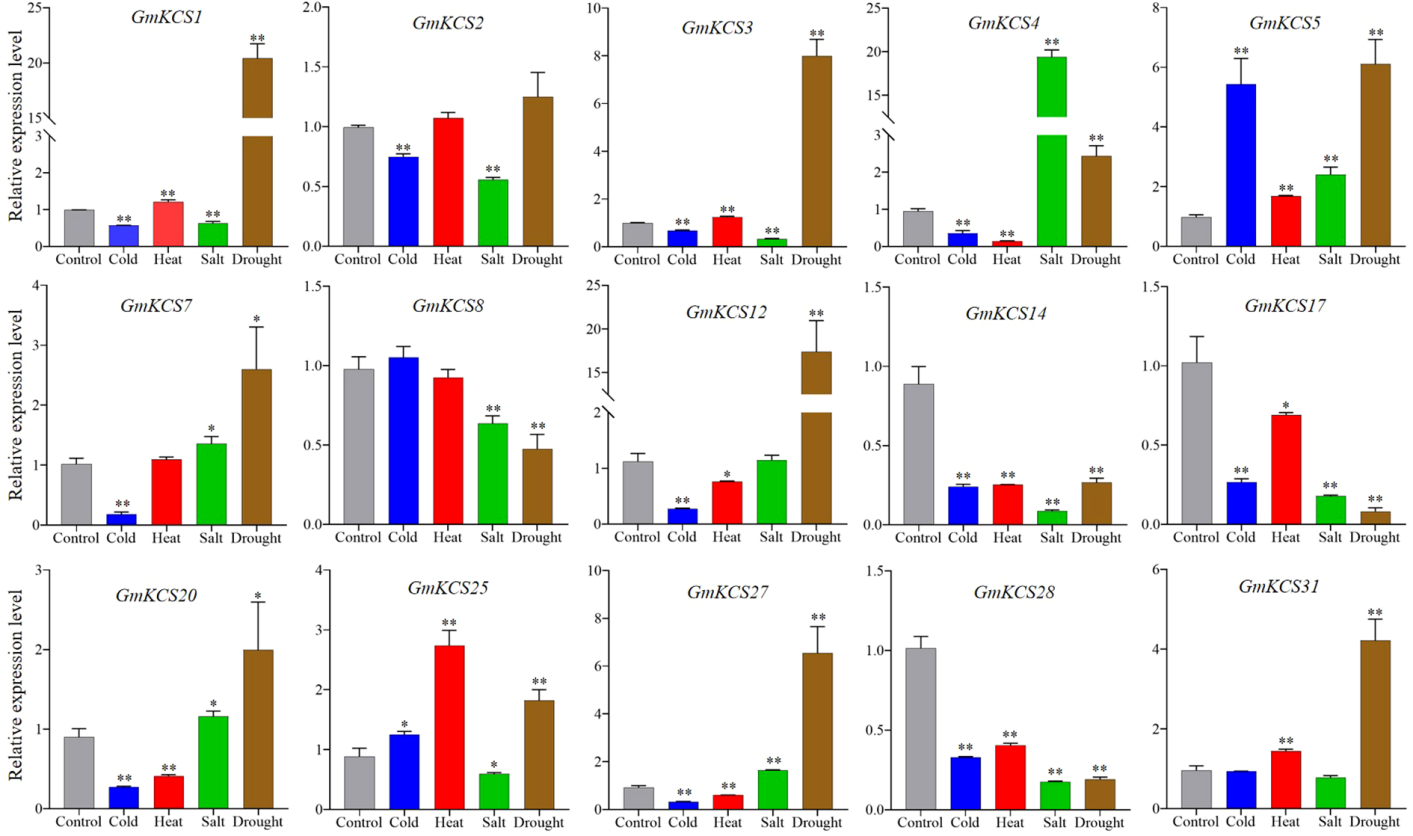
Relative expression levels of *GmKCS* genes under heat, cold, salt, and drought stress in soybean. qRT-PCR was used to study the expression levels of fifteen leaf-expressed *GmKCS* genes in triplicates. Error bars indicate SE and p-value < 0.05 (*) or < 0.01 (**).

To further elucidate the putative functions of *GmKCS* genes in response to abiotic stresses, we examined, using qRT-PCR, the expression patterns of 15 leaf-expressed *GmKCS* genes in soybean leaves subjected to cold, heat, salt, and drought, 24 h after the stress treatments. A greater than two-fold change in the transcriptional levels of a gene in any of the treated samples relative to that of the control and *P* < 0.05 was considered to be significantly differentially expressed. As shown in **Figure 6**, *GmKCS*s *4*, *14*, and *28* were differentially expressed under all four abiotic stresses, indicating that they may be essential for enhancing soybean plants’ tolerance under stress conditions. Among the three genes, *GmKCS14* and *GmKCS28* were significantly down-regulated under all four stress conditions. *GmKCS4* was significantly up-regulated by salt and drought stress, while it was repressed under cold- and heat-induced stress. Under cold stress, *GmKCS*s *4*, *7*, *12*, *14*, *17*, *20*, *27*, and *28* were down-regulated. The relative expression of *GmKCS5* was induced by 4.77-fold compared to the control, while the other 6 genes were not differentially expressed. Under heat stress, the expression level of *GmKCS*s *4*, *14*, *20*, and *28* significantly decreased. Specifically, the relative expression of *GmKCS25* increased by 1.74-fold compared to the control, while the expression of the remaining genes did not significantly change. Under salt stress, *GmKCS4* and *GmKCS5* were up-regulated, with *GmKCS4* being drastically up-regulated by 18.39-fold compared to the control. In contrast, *GmKCS*s *3*, *14*, *17*, and *28* showed varying degrees of down-regulation, and the expression level of the other 9 *GmKCS* genes did not significantly change under salt stress. Except for *GmKCS2* and *GmKCS25*, the expression level of the other 13 *GmKCS* genes was significantly altered under drought stress, among which 9 and 4 genes were up- and down-regulated, respectively. Notably, the expression level of *GmKCS*s *1*, *3*, *5*, *12*, and *27* under drought stress showed greater than 5-fold up-regulation compared with the control.

## Discussion

Cuticular wax forms the first protective barrier for the aerial parts of all terrestrial plants and plays a vital role in plant resistance against various biotic and abiotic stresses (Kong et al., 2020; Batsale et al., 2021). VLCFAs and their derivatives, including alkanes, ketones, alcohols, aldehydes, and wax esters, are the main components of cuticular wax. Thus, their content and composition are tightly associated with plant growth, development, and stress tolerance capability (Haslam and Kunst, 2013; Scott et al., 2022). In Arabidopsis, drought, salt and abscisic acid stress treatments lead to a significant increase in the amount of leaf cuticular wax (Kosma et al., 2009). Total leaf cuticular wax of tree tobacco (Nicotiana glauca) increased approximately 2-fold under drought stress, and leaves subjected to drying stress were more resistant to water loss than that of control (Cameron et al., 2006). Various stress conditions can elicit diverse impacts on the content, composition, and structure of plant cuticular wax (Shepherd and Wynne, 2006). The systematic identification and analysis of genes involved in plant cuticular wax synthesis and their expression patterns under stress are of great significance for understanding the molecular regulatory mechanisms of plant response to stress.

3-ketoacyl-CoA synthases, encoded by the *KCS* gene family, have been confirmed to be the key rate-limiting enzymes that determine the tissue and substrate specificities during VLCFA production, which are present in nearly all plants (Ozseyhan et al., 2018). The genome-wide identification and functional characterization of the *KCS* gene family have been described in many plant species, and large differences have been observed in the number of *KCS* family genes (ranging from 7 to 58) in higher plants (Guo et al., 2016; Campbell et al., 2019; Lian et al., 2020; Xue et al., 2020; Zhang et al., 2022a). However, our understanding of the structural and functional properties of the *KCS* gene family in soybean, one of the most important food and oil crops worldwide, is greatly limited. Identification and characterization of the soybean *KCS* family genes at the whole genome level could provide fundamental information for their functions and facilitate the genetic improvement of soybean.

In this study, a total of 31 *KCS* genes were identified in the soybean genome (**Table 1**; **Figure 1**), which is the highest number among all the diploid plants analyzed to date (Joubès et al., 2008; Guo et al., 2016; Campbell et al., 2019; Zhang et al., 2022a; Yang et al., 2023). A higher gene number occurrence has also been reported for other gene families in soybean, such as the B-box (Shan et al., 2022), the cytokinin oxidase (Du et al., 2023), and the actin-depolymerizing factor gene families (Sun et al., 2023). The *KCS* gene family expansion observed in soybean is possibly due to the two whole genome duplication events that occurred during soybean evolution (Schmutz et al., 2010). It appears that the *KCS* family genes have been significantly subjected to duplication events and expansion in soybean. Tandem and segmental duplications are considered to be the two main modes of gene family expansion during species evolution (Lynch and Conery, 2000). The former often results in a cluster of genes in the same or adjacent intergenic regions (Lynch and Conery, 2000), which has been observed among *KCS* genes in many species, such as Arabidopsis (Joubès et al., 2008), apple (Lian et al., 2020), sorghum (Zhang et al., 2022a) and barely (Tong et al., 2021). However, segmental duplications are the main driving force in *KCS* gene family expansion. In this study, a synteny analysis showed that 27 duplicated gene pairs covering 28 *GmKCS* genes were found to be generated by segmental duplication (**Figure 1**), which was also supported by their similar exon-intron structures (**Figure 3**). However, no tandem duplication event was identified as responsible for the expansion of the *GmKCS* family in soybean. Therefore, segmental duplications were primarily responsible for the *KCS* family’s expansion during soybean evolution. Moreover, these duplicated gene pairs were not only clustered into the same branches of the phylogenetic tree but also exhibited low Ka/Ks ratios (**Figure 1**; **Figure 2**; **Supplementary Table S3**), implying that *GmKCS* genes underwent a strong purifying selection during evolution and their functions might be evolutionarily conserved.

Generally, genes clustered in the same evolutionary clade are thought to perform similar biological functions (Liu et al., 2018). Based on their phylogenetic relationships, 131 KCS proteins from *Arabidopsis*, turnip, soybean, rice and sorghum were clustered into nine groups (**Figure 2**), consistent with previously published reports (Joubès et al., 2008; Xiao et al., 2016; Xue et al., 2020; Yang et al., 2023). Group λ and η *KCS* genes are unique to monocotyledonous and dicotyledonous plants, respectively, suggesting that these genes might be formed after the separation of monocots and dicots and functions between these genes may have differentiated (**Figure 2**; **Table 2**). Moreover, we found that KCSs in group β only existed in in two cruciferous plants, *Arabidopsis* and turnip. To explore whether group β *KCS* genes have been lost only in soybean, rice and sorghum, we searched for articles on the *KCS* gene family in other plant species. Interestingly, the loss of the β group *KCS* gene was also reported in cotton (Xiao et al., 2016), apple (Lian et al., 2020), peanut (Huai et al., 2020), barely (Tong et al., 2021), yellow horn (Liu et al., 2022), sorghum (Zhang et al., 2022a), passion fruit (Rizwan et al., 2022), and rice (Yang et al., 2023). Consistent with our results, *KCS* genes in group β have also been found in rapeseed and cabbage (Xue et al., 2020), implying that group β *KCS* genes may be unique to cruciferous plants. Genes in the other six groups might have undergone similar evolutionary diversification across plant families and species. The *GmKCS* gene homologs of known function in Arabidopsis provided clues or references for predicting and exploring the biological function of *GmKCS* genes (**Figure 2**). For example, the group α *GmKCS* genes were clustered with *AtKCS4* and *AtKCS9*, which are widely involved in the biosynthesis of cuticular wax, suberin, sphingolipids, and phospholipids (Kim et al., 2013; Kim et al., 2021). *GmKCS2* in the group δ is closely related to *AtKCS1*, which has been shown to regulate cuticular wax biosynthesis and tolerance to chilling stress in *Arabidopsis* (Todd et al., 1999; Chen et al., 2020c). The group ζ *GmKCS* genes are closely related to *AtKCS2* and *AtKCS20*, which are involved in the biosynthesis of cuticular wax and root suberin and tolerance to osmotic stress (Lee et al., 2009; Lee and Suh, 2015a), *GmKCS3* and *GmKCS8*, classified in group ε, are closely related to *AtKCS10*, which is required for normal development of the epidermis (Pruitt et al., 2000).

FAE1_CUT1_RppA and ACP_syn_III_C are the two major conserved domains in KCS proteins. The former had been reported to contain the motif that specifically binds to the substrate and thus may be related to the substrate specificity of each KCS enzyme, whereas the latter is important for initiating the fatty acid synthase chain reactions (Funa et al., 2002; Sagar et al., 2015). Our motif analysis showed that although the ACP_syn_III_C domain is conserved among GmKCS proteins, diverse sequence variants were found in the FAE1_CUT1_RppA domain (**Figure 3C**). Moreover, we also found the N-terminal was poorly conserved among GmKCS proteins. For example, there is a significant difference in sequence length before the FAE1_CUT1_RppA domain, and some of the GmKCS contain motifs 6 and 7. In contrast, other gene family members don’t contain any of the motifs (**Figure 3C**). The different distribution of motifs within KCS proteins was also reported in previous studies (Lian et al., 2020; Liu et al., 2022; Rizwan et al., 2022). Differences in GmKCS protein sequence characteristics imply that functional differentiation through evolution may have occurred, and further research on these sequence variations is needed for their functional elucidation and exploration.

*Cis*-acting elements play critical roles in signal transduction and regulation of gene transcription initiation (Narusaka et al., 2003). The mechanisms underlying the expression regulation of each *KCS* gene could be better understood by identifying the upstream cis-acting elements of the *GmKCS* genes. In agreement with previous reports concerning the *KCS* gene family (Lian et al., 2020; Rizwan et al., 2022; Zhang et al., 2022a), *cis*-acting elements involved in hormone- and stress-responsiveness were found to be widely distributed in *GmKCS* promoters, and all *GmKCS* promoters contain at least one type of hormone- and stress-responsive elements (**Figure 4**). Previous studies demonstrated that these elements are involved in gene expression regulation under various stresses (Jiang et al., 2022; Dong et al., 2023; Zhang et al., 2023). Our results suggest that *GmKCS* expression is closely associated with abiotic stress and hormone-signaling responses.

The diverse expression patterns of *KCS* genes in different tissues have been described in many plants. For example, all 21 *AtKCS* genes were found to be expressed but had diverse expression levels and tissue distributions (Joubès et al., 2008). Furthermore, certain rice *KCS* genes exhibit specific expression in different organs or different stages of growth and development (Yang et al., 2023). Exploring the expression profiles of *GmKCS* genes may help uncover their potential roles in plant physiological processes and development. We obtained the expression patterns of each *GmKCS* gene in nine tissues/organs from a public database. 27 out of the 31 *GmKCS* genes were found to be expressed in at least one tissue/organ, and six of which (i.e., *GmKCSs* 2, 5, 7, 20, 25 and 27) were differentially expressed throughout the different organs of the soybean plants (**Figure 4**; **Supplementary Table S3**). Several members that were constitutive expressed throughout the plants (*GmKCSs* 2, 5, 7, 20, 25, and 27) or predominantly expressed in specific tissue/organs (*GmKCSs* 6, 15, 17, and 26) were identified (**Figure 4**; **Supplementary Table S3**). The differential expression of *GmKCS* genes in different tissues/organs, and for certain genes, their tissue specificity, may indicate that they perform different functions in different plant organs and remains to be further investigated.

Unfavorable abiotic stress environmental conditions are often detrimental to growth and development. Drought, salt, and temperature stresses are the main environmental factors that limit the productivity of agricultural plants and threaten food security (Zhang et al., 2022b). Increasing evidence suggests that plant *KCS* genes are involved in abiotic stress responses and adaptation (Batsale et al., 2021). For instance, Arabidopsis kcs1 mutants exhibited reduced resistance to low humidity conditions at a young age (Todd et al., 1999), while overexpression of drought-induced *KCS* genes, such as *CsKCS6* from orange, *BnKCS1* from rapeseed, or *AhKCS1* from groundnut, enhanced the drought tolerance of transgenic plants (Lokesh et al., 2019; Guo et al., 2020; Wang et al., 2020b). These genes can be targeted in crop breeding to improve drought tolerance. In our study, we examined the transcriptional profiles of the 15 leaf-expressed *GmKCS* gene family members under four abiotic stress conditions. Our qRT-PCR results showed that the expression levels of 9, 5, 6, and 13 *GmKCS* genes were altered more than two-fold under cold, heat, salt, and drought stress, respectively (**Figure 6**). Some of them exhibited a greater than 5 fold up-regulation under stress conditions, i.e., *GmKCS5* under cold stress, *GmKCS4* under salt stress, and *GmKCSs* 1, 3, 5, 12, and 27 under drought stress (**Figure 6**). The results indicated that the *GmKCS* genes may widely be involved in abiotic stress adaptation and resistance of soybean. Currently, there is limited evidence regarding the potential link between the differential expression of *GmKCS* genes and the accumulation of cuticular wax in leaves, as well as the response to stress in soybean. Further research is required to investigate the alterations in VLCFAs content and components in soybean plants following stress treatment. Additionally, it is important to identify the specific *KCS* genes that are instrumental in regulating soybean stress resistance. By targeting these key *GmKCS* genes, soybean breeding practices can enhance the plant’s tolerance to stress.

## Conclusions

In this study, a total of 31 *KCS* genes were identified in the soybean genome, and their physicochemical properties, chromosomal location, collinearity, evolutionary relationships, gene structure, the presence of motifs and *cis*-acting elements, and gene expression patterns in different tissues/organs and under abiotic stress conditions were analyzed. The *GmKCS* genes were distributed on 14 chromosomes, and segmental duplication during evolution was primarily responsible for their expansion during soybean genome evolution. *GmKCS* genes could be divided into seven groups based on their phylogenetic relationships. *GmKCS* genes exhibited diverse expression patterns in the various tissues/organs of soybean plants. Many of them were found to respond to varied abiotic stresses, implying that they may be crucial for stress adaptation and tolerance in soybean. These findings will contribute to future research regarding the functions and regulatory mechanisms underlying soybean *KCS* genes in response to abiotic stress and their potential exploitation towards improving soybean stress tolerance.

## Data availability statement

The original contributions presented in the study are included in the article/**Supplementary Material**. Further inquiries can be directed to the corresponding authors.

## Author contributions

YG: Investigation, Methodology, Writing – original draft. DW: Investigation, Software, Writing – original draft. HX: Conceptualization, Formal Analysis, Writing – original draft. ZZ: Investigation, Writing – original draft. YC: Investigation, Writing – original draft. DZ: Investigation, Writing – original draft. YJ: Investigation, Writing – original draft. MS: Software, Writing – original draft. PL: Software, Writing – original draft. QS: Software, Writing – original draft. JY: Investigation, Software, Writing – original draft. PC: Writing – original draft. YS: Investigation, Methodology, Software, Writing – original draft, Writing – review & editing.

## Supplementary material

Supplementary Data Sheet 1 KCS protein sequences from five species used for phylogenetic analysis.
