# Supplementary material for: Genome-wide identification and expression analysis of the KCS gene family in soybean (Glycine max) reveal their potential roles in response to abiotic stress
Source: Front Plant Sci. 2023 Dec 5;14:1291731. doi: 10.3389/fpls.2023.1291731 (PMC10728876; doi:10.3389/fpls.2023.1291731)
Supplement: Supplementary Data Sheet 1 — KCS protein sequences from five species used for phylogenetic analysis. [file DataSheet_1.pdf]

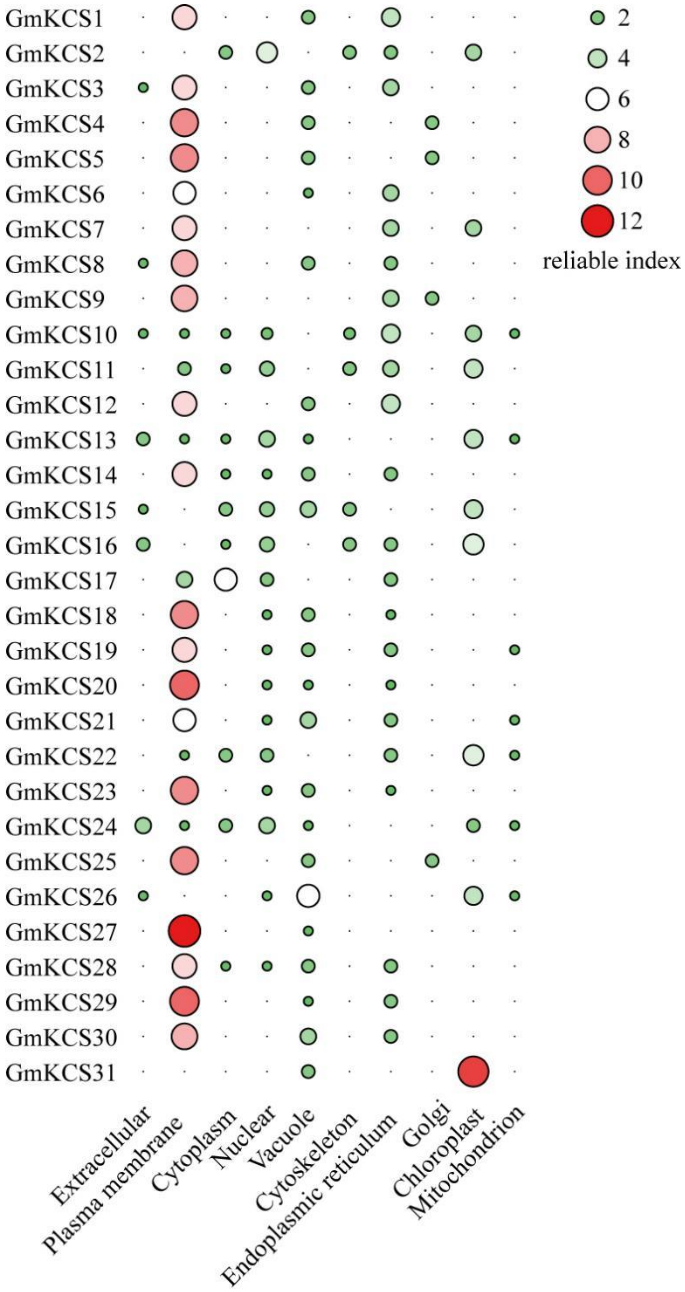

Figure S1 The subcellular localization prediction for GmKCS proteins. The color and the size of the circles indicate the reliability of the prediction results. The name of each protein is shown on the left. The site name for the predicted subcellular localization of each GmKCS protein is shown at the bottom.

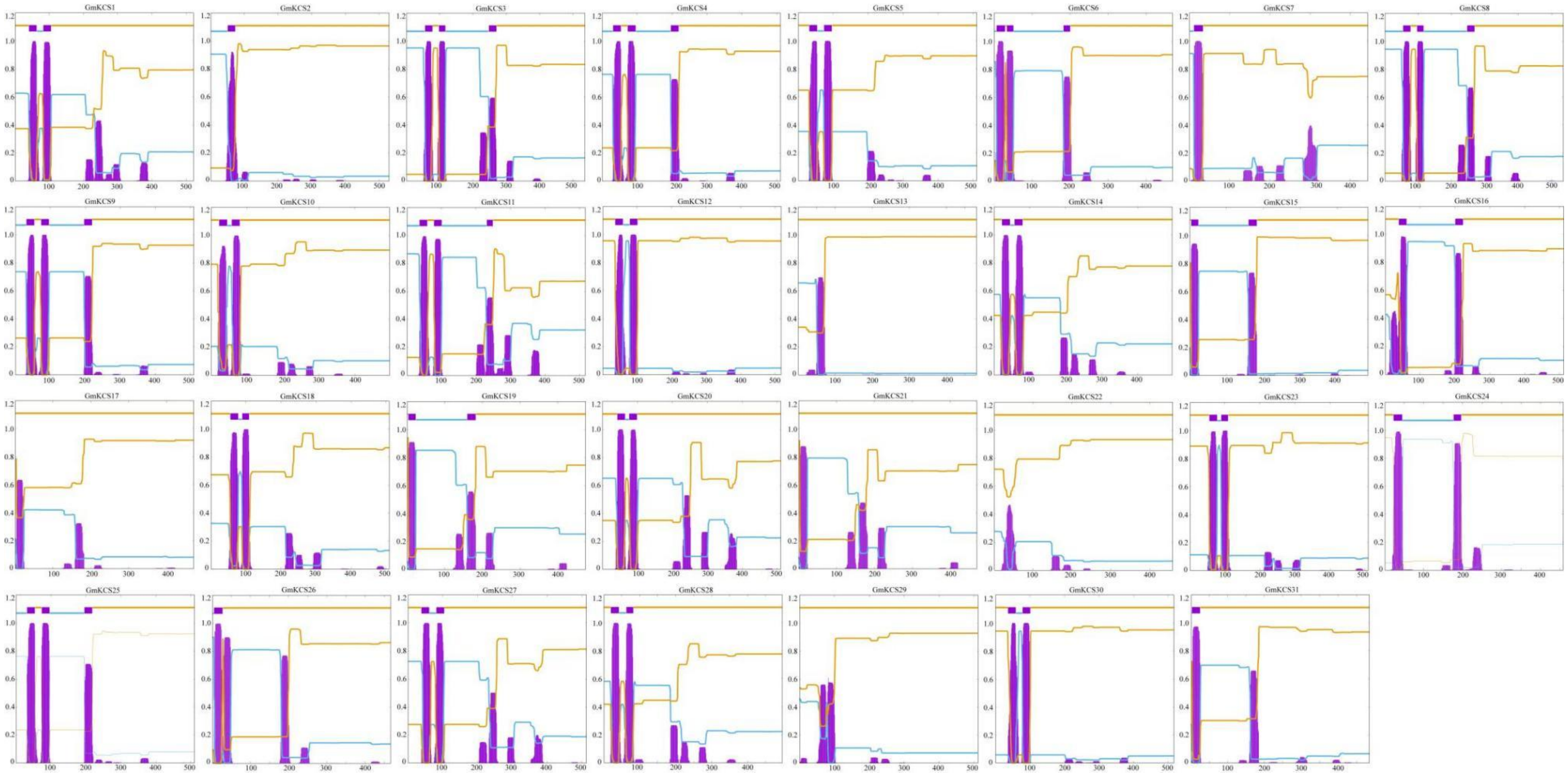

Figure S2 Transmembrane alpha helix structure of GmKCS proteins. The red rectangles represent the transmembrane alpha helixes, the blue thickened lines represent the amino acids in this segment that are located inside the membrane, the pink thickened lines represent the amino acids in this segment that are located outside the membrane, the scale on the left side indicates the probability of correct prediction.

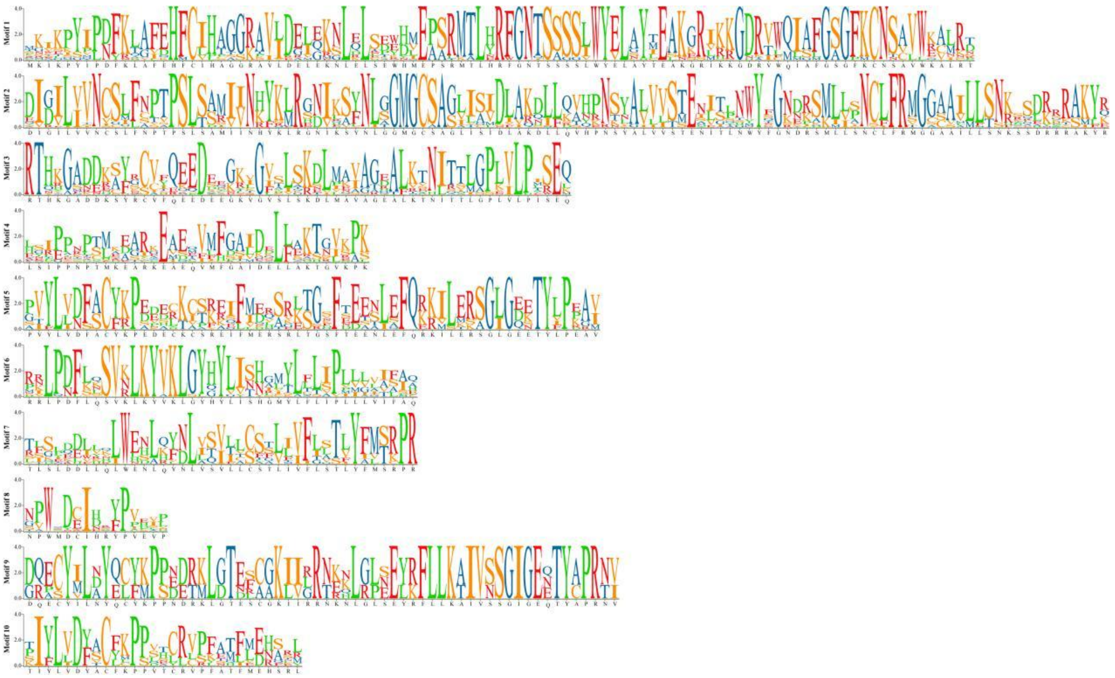

Figure S3 The sequences information of 10 conserved motifs identified in GmKCS proteins.

Table S1 The sequences of the primers used for the qRT-PCR analysis.

Gene name	Forward primer	Reverse primer
Tubulin	TCTTGGACAACGAAGCCATCT	TGGTGAGGGACGAAATGATCT
GmKCS1	TTCTAGGCATAGCTTCGGCTC	ACACCGTGAGGCTTGAACAT
GmKCS2	CGGTGTACCTGGTGGACTTC	GAACATGACTGCCTCTGCCT
GmKCS3	GCTGGCTCGTTGACATTCTCT	AAGAACCGTAGCAAGGTCGT
GmKCS4	GCTCTCGAGCTTCTCCCTTG	CCCTCTCCAGAATCTTCCGC
GmKCS5	TCTCGGAAGTGTGCGAAAAGG	TGTTCTCCCCAAGGCCAGAT
GmKCS7	AATTGCCTTTTCCGCGTTGG	CCGACCTCGTCTTGTTCCTG
GmKCS8	CGGCGACGAGACCTACATC	GGTGTCGGGTTGAAGATGCT
GmKCS12	TGTCTCAGTGACTCTATGTTCTAGC	TACTCAAGATTGCTGGGGGC
GmKCS14	CTTGCCGGATTTCTCCAACTC	GAAGGCGGAGCAGAGGATTT
GmKCS17	CTTCCGAACCGGGGTTGAT	CAATCGTCCCACACATTCGC
GmKCS20	TCTCTCAGACCACCGACCTC	CTGGAACTCCAGGGAGGACT
GmKCS25	ACAGCTGCTATTCTTTGCCAC	TGCTGGAGGTGTTTCCGAAT
GmKCS27	TTCAGACCACCGACATCGAC	GTGACGGCGTAGAGAGTGAG
GmKCS28	GCAGGACTTGTCAAACTCGG	GGCCTAAGCGTAGAACCTCG
GmKCS31	CGTCCCTCGGAGGTAGACAT	CACCGAGATCCTCAGTGCTC

Table S2 List of the protein IDs of HMMER and BLASTP searches.

Results of HMMER search	Results of BLASTP search	Integrated Results
Glyma.01G073600.1	Glyma.01G073600.1	Glyma.01G073600.1
Glyma.01G091400.1	Glyma.01G091400.1	Glyma.01G091400.1
Glyma.01G228700.1	Glyma.01G228700.1	Glyma.01G228700.1
Glyma.02G001500.1	Glyma.02G001500.1	Glyma.02G001500.1
Glyma.02G130400.1	Glyma.02G130400.1	Glyma.02G130400.1
Glyma.03G260300.1	Glyma.02G266400.1	Glyma.02G266400.1
Glyma.04G057800.1	Glyma.03G260300.1	Glyma.03G260300.1
Glyma.04G149300.1	Glyma.04G057800.1	Glyma.04G057800.1
Glyma.05G011100.1	Glyma.04G149300.1	Glyma.04G149300.1
Glyma.05G083000.1	Glyma.05G011100.1	Glyma.05G011100.1
Glyma.05G153200.1	Glyma.05G083000.1	Glyma.05G083000.1
Glyma.06G012500.1	Glyma.05G153200.1	Glyma.05G153200.1
Glyma.06G058500.1	Glyma.06G012500.1	Glyma.06G012500.1
Glyma.06G118550.1	Glyma.06G058500.1	Glyma.06G058500.1
Glyma.06G214800.1	Glyma.06G118550.1	Glyma.06G118550.1
Glyma.08G109200.1	Glyma.06G214800.1	Glyma.06G214800.1
Glyma.08G109300.1	Glyma.08G109200.1	Glyma.08G109200.1
Glyma.08G109400.1	Glyma.08G109300.1	Glyma.08G109300.1
Glyma.08G109500.1	Glyma.08G109400.1	Glyma.08G109400.1
Glyma.08G110300.1	Glyma.08G109500.1	Glyma.08G109500.1
Glyma.08G110340.1	Glyma.08G110300.1	Glyma.08G110300.1
Glyma.08G110420.1	Glyma.08G110340.1	Glyma.08G110340.1
Glyma.08G110460.1	Glyma.08G110420.1	Glyma.08G110420.1
Glyma.08G110500.1	Glyma.08G110460.1	Glyma.08G110460.1
Glyma.08G110701.1	Glyma.08G110500.1	Glyma.08G110500.1
Glyma.08G110801.1	Glyma.08G110701.1	Glyma.08G110701.1
Glyma.08G110901.1	Glyma.08G110801.1	Glyma.08G110801.1
Glyma.08G261100.1	Glyma.08G110901.1	Glyma.08G110901.1
Glyma.09G043700.1	Glyma.08G261100.1	Glyma.08G261100.1
Glyma.09G075200.1	Glyma.09G043700.1	Glyma.09G043700.1
Glyma.10G001800.1	Glyma.09G075200.1	Glyma.09G075200.1
Glyma.10G179400.1	Glyma.10G001800.1	Glyma.10G001800.1
Glyma.10G241700.1	Glyma.10G179400.1	Glyma.10G179400.1
Glyma.10G274400.1	Glyma.10G241700.1	Glyma.10G241700.1
Glyma.10G291700.1	Glyma.10G274400.1	Glyma.10G274400.1
Glyma.11G011500.1	Glyma.10G291700.1	Glyma.10G291700.1
Glyma.11G097900.1	Glyma.11G011500.1	Glyma.11G011500.1
Glyma.11G144809.1	Glyma.11G097900.1	Glyma.11G097900.1
Glyma.12G075100.1	Glyma.11G144809.1	Glyma.11G144809.1
Glyma.13G238600.2	Glyma.12G075100.1	Glyma.12G075100.1
Glyma.13G331600.1	Glyma.13G238600.2	Glyma.13G238600.2

Glyma.14G074300.1	Glyma.13G331600.1	Glyma.13G331600.1
Glyma.15G042500.1	Glyma.14G074300.1	Glyma.14G074300.1
Glyma.15G046300.1	Glyma.15G042500.1	Glyma.15G042500.1
Glyma.15G074700.1	Glyma.15G046300.1	Glyma.15G046300.1
Glyma.15G149400.1	Glyma.15G074700.1	Glyma.15G074700.1
Glyma.17G118700.1	Glyma.15G149400.1	Glyma.15G149400.1
Glyma.17G183700.1	Glyma.17G118700.1	Glyma.17G118700.1
Glyma.17G251000.1	Glyma.17G183700.1	Glyma.17G183700.1
Glyma.19G105100.1	Glyma.17G225450.1	Glyma.17G225450.1
Glyma.20G115500.1	Glyma.17G251000.1	Glyma.17G251000.1
Glyma.20G152500.1	Glyma.20G115500.1	Glyma.19G105100.1
Glyma.20G210900.1	Glyma.20G152500.1	Glyma.20G115500.1
Glyma.20G240900.1	Glyma.20G210900.1	Glyma.20G152500.1
	Glyma.20G240900.1	Glyma.20G210900.1
		Glyma.20G240900.1

Table S3 The *Ka*/*Ks* ratios of the duplicated *GmKCS* gene pairs.

Duplicated gene pairs	Ka	Ks	Ka/Ks	Time (Mya)
GmKCS7/GmKCS20	0.151853026	1.728804824	0.087836998	141.71
GmKCS7/GmKCS27	0.145810561	1.744662523	0.083575224	143.01
GmKCS20/GmKCS27	0.014744621	0.19045795	0.077416672	15.61
GmKCS10/GmKCS14	0.054581897	1.264002729	0.043181787	103.61
GmKCS10/GmKCS28	0.054619076	1.304735425	0.041862185	106.95
GmKCS14/GmKCS28	0.014160757	0.211396732	0.066986641	17.33
GmKCS1/GmKCS11	0.02786831	0.143799066	0.193800357	11.79
GmKCS1/GmKCS30	0.059858111	0.784088284	0.076341035	64.27
GmKCS11/GmKCS12	0.07657563	0.719976673	0.106358487	59.01
GmKCS12/GmKCS30	0.008446035	0.123400759	0.068443949	10.11
GmKCS4/GmKCS5	0.065214781	0.818786782	0.079648063	67.11
GmKCS4/GmKCS9	0.007704997	0.131735665	0.058488318	10.80
GmKCS4/GmKCS25	0.067984534	0.788754198	0.086192294	64.65
GmKCS5/GmKCS9	0.074106607	0.784156712	0.094504843	64.28
GmKCS5/GmKCS25	0.015619915	0.13773339	0.113406889	11.29
GmKCS9/GmKCS25	0.075011978	0.786400625	0.095386467	64.46
GmKCS6/GmKCS26	0.019707108	0.279606859	0.07048149	22.92
GmKCS3/GmKCS8	0.006099661	0.208763929	0.029217983	17.11
GmKCS18/GmKCS23	0.028471365	0.13642411	0.208697459	11.18
GmKCS13/GmKCS29	0.031444846	0.107354778	0.292905881	8.80
GmKCS15/GmKCS31	0.018871746	0.132022468	0.142943442	10.82
GmKCS16/GmKCS17	0.020475911	0.225213557	0.090917755	18.46
GmKCS16/GmKCS19	0.171369901	1.751005056	0.097869449	143.53
GmKCS16/GmKCS21	0.156686148	1.63226433	0.095993121	133.79
GmKCS17/GmKCS19	0.171738868	1.667181129	0.103011524	136.65
GmKCS17/GmKCS21	0.167266833	1.705718688	0.098062379	139.81
GmKCS19/GmKCS21	0.016071634	0.15171596	0.105932386	12.44

Table S4 FPKM values of *GmKCS* genes in nine soybean tissues/organs.

Name	roots	root hairs	nodules	stems	leaves	SAM	flowers	pods	seeds
GmKCS20	1.72	3.30	2.01	4.92	4.14	1.10	1.91	6.26	5.09
GmKCS27	30.75	17.43	13.44	11.55	7.51	5.22	6.11	20.96	13.31
GmKCS7	10.42	6.36	3.54	6.68	2.47	3.07	1.07	3.69	8.15
GmKCS10	0.53	0.03	0.00	0.07	0.32	3.54	1.24	1.14	0.06
GmKCS14	15.62	3.13	0.13	52.88	83.25	35.70	35.76	47.69	0.26
GmKCS28	9.92	1.24	0.10	15.44	18.62	51.76	23.79	25.15	0.97
GmKCS2	2.95	2.72	1.15	1.26	3.15	2.66	3.98	7.06	4.70
GmKCS1	6.59	1.40	0.97	3.59	5.47	12.72	6.05	1.82	0.00
GmKCS11	5.43	1.23	0.76	3.42	0.65	6.00	0.68	1.12	0.00
GmKCS12	12.98	5.25	3.09	5.67	9.15	28.62	9.90	3.04	0.79
GmKCS30	10.17	3.50	2.60	3.27	0.48	15.33	7.36	2.45	0.09
GmKCS4	17.53	7.03	4.31	0.39	7.70	1.03	0.87	0.29	0.71
GmKCS9	5.37	2.12	1.36	0.12	1.18	0.51	0.32	0.28	0.88
GmKCS5	11.66	14.51	9.68	10.01	12.09	8.93	4.06	9.47	5.10
GmKCS25	27.32	15.11	10.08	9.73	14.43	24.91	6.31	9.98	8.89
GmKCS6	11.98	0.00	0.00	2.63	0.00	98.07	12.57	0.00	0.05
GmKCS26	10.94	0.02	0.00	2.37	0.00	76.31	16.02	0.05	0.02
GmKCS3	33.45	4.54	0.24	26.07	49.84	166.89	55.59	34.00	0.94
GmKCS8	22.91	2.67	0.20	11.18	5.12	126.29	22.19	13.64	7.93
GmKCS18	0.35	0.27	1.80	0.08	0.03	0.04	3.41	0.01	0.00
GmKCS23	0.00	0.02	0.00	0.29	0.00	0.00	0.58	0.08	0.00
GmKCS22	0.24	0.08	0.28	0.05	0.03	0.01	0.37	0.04	0.02
GmKCS24	0.00	0.00	0.00	0.00	0.00	0.00	0.31	0.00	0.00
GmKCS16	1.36	0.00	0.00	1.79	0.69	11.88	1.22	2.26	1.45
GmKCS17	1.20	0.00	0.00	1.47	39.29	8.35	12.56	0.71	0.00
GmKCS19	8.03	0.68	1.05	1.64	0.03	6.34	1.70	1.01	0.01
GmKCS21	6.41	0.74	0.52	1.84	0.01	6.86	3.73	0.34	0.00
GmKCS15	5.04	0.30	0.08	6.07	0.28	16.36	1.97	3.33	0.08
GmKCS31	4.65	0.00	0.23	26.68	5.51	9.23	20.1	12.17	0.05
GmKCS13	0.94	0.61	0.35	0.03	0.27	0.37	2.06	0.82	5.44
GmKCS29	0.45	0.24	0.00	0.05	0.00	0.65	0.62	0.18	0.28
